# Engineering Approaches to Prevent Blood Clotting from Medical Implants

**DOI:** 10.33552/abeb.2018.01.000510

**Published:** 2019-03-29

**Authors:** Aaron C Wilson, Pierre F Neuenschwander, Shih-Feng Chou

**Affiliations:** 1 Department of Mechanical Engineering, The University of Texas at Tyler, USA; 2 Department of Cellular and Molecular Biology, The University of Texas Health Science Center at Tyler, USA

**Keywords:** Blood clotting, Vascular stents, Polymer coatings, Surface properties

## Abstract

Injectable and/or Implantable medical devices are widely used in the treatment of diseases. Among them, vascular stents provide the medical solution to treat blood clotting. However, traditional metallic stents, even with current improvements in anticoagulation properties, have potential drawbacks in local inflammation when first implanted into the body and undesirable protein adsorption and cell adhesion after a prolonged period of time in the body. In this perspective, we discuss several engineering approaches, including drug-eluting materials, polymeric and non-polymeric coatings, and surface modifications to coating materials that can be applied to the surface of medical implants to significantly improve the hemocompatibility. These coatings are expected to have a slow degradation rate with the ability to either load drugs or attach biomacromolecules to form an architecture that mimics the surrounding cells. In general, our perspective provides a current view on the achievements of hemo-compatible coatings and future trends in coating materials that will extend the life of the medical implants.

## Introduction

The use of injectable and/or implantable medical devices has emerged as one of the most attractive engineering approaches toward the treatment of many diseases [1]. These medical implants not only provide structural support to the surrounding tissues but also can be integrated with medical functions, such as drug release or data recording/transmission, just to name a few. To fully capture the mechanical and biomedical aspects of these implantable medical devices, one important task is to minimize the inflammation response generated by the immune system. Through engineering approaches, a particular method that has been widely utilized is to coat the surface of the medical implants with a more biocompatible material that reduces the risk of device rejection [2].

One of the most widely used implantable medical devices is vascular heart stent for thrombosis treatment, where 1.8 million stent implant procedures were made each year in the US [3]. Traditionally, these vascular stents have been made from thromboresistant metals. However, deposition of proteins and local inflammation leading to an immune response prohibit the long-term use of these metallic stents. Due to the degradation of their medical performance, previous efforts have been focused on the development of polymeric coatings with drug release mechanisms that reduce the inflammatory response when stents are initially placed in the body. However, adverse side effects from this approach were noticeable due to the depletion of drug as well as cytotoxicity as a result of the enhanced biodegradation rate accompanied with drug release. In addition, the conventional polymeric coatings exhibited poor surface properties that result in undesirable protein adsorption and cell adhesion. Based on these observations, the focus of the coating materials has translated into the design of a more desirable coating surface with properties that can mimic the surrounding cells/tissues [4–6].

In this perspective, we provide a summary of the blood clotting cascade and the types of coating materials that are currently being used for anticoagulation purposes on vascular stents. These techniques range from drug-eluting materials, polymeric and non-polymeric coatings, and surface modifications of the coatings. In particular, surface modifications of polymeric coatings utilize chemical grafting procedures where covalently bonded biomacromolecules are assembled into architectures mimicking local cells for better therapeutic performance. Grafting is a process to attach molecules onto the surface of an object, and its advantages include the easy introduction, controllable density, and exact localization of graft chains at the surface, without changing the bulk properties of the materials. In some cases, graft chains via covalent bonding onto polymer surfaces are necessary to avoid molecule delamination with long-term chemical stability. Overall, our perspective provides an update and our views on current technologies and trends in thromboresistant medical implants, which aim to prolong the device life to achieve the desired therapeutic performance as a medical implant.

## Factors to Consider

### Clotting cascade

**Figure 1:**
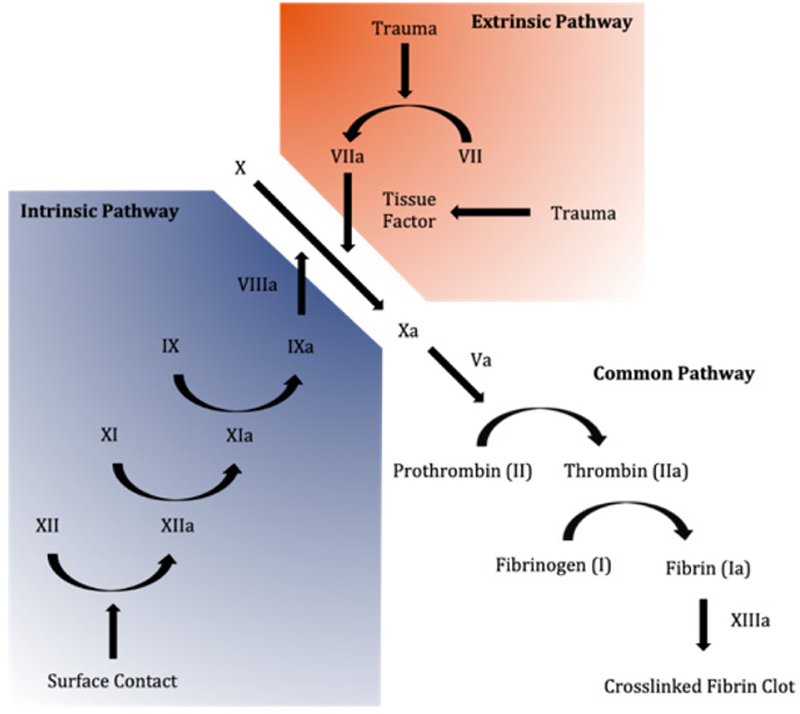
Schematic illustration of the blood clotting cascade.

In order to determine whether a polymeric coating will be effective for anticoagulation, it is important to first understand how the clotting process works when the body is introduced to these materials Figure 1 [7]. There are two ways by which the clotting process is initiated, the tissue factor pathway and the contact pathway. The contact pathway is initiated when the blood comes into contact with foreign objects, such as the implantable medical devices, which activates a plasma protein called factor XII. After a mass activation (or cascade) of other enzymes and proteins in the blood that triggers IXa [8], it concludes with the polymerization of the protein fibrin and the activation of platelets, which together form blood clots [9].

The reason for this activation is that, under normal circumstances, blood is only in contact with endothelial cells that cover the walls of the vessels throughout the circulatory system. These cells, when healthy, provide a number of important uses, including preventing coagulation. When these cells are damaged, the surface where the damage occurred becomes thrombotic and the clotting cascade is initiated [10] (Figure 1).

Since implantable devices do not have an endothelial layer covering them, they are essentially seen as a damaged vessel surface and the clotting process will initiate in an attempt to fix the damage. However, instead of the clots plugging a hole in the wall of a vessel, they will be formed around the surface of the biomaterial. This could not only cause an implanted device to fail, but it could also cause the flow of blood to be cut off entirely in vessels and arteries, which could be very damaging or even fatal to the patient.

### Biocompatibility

Another important factor to consider when considering biomaterials for anticoagulation is the biocompatibility of the material. The main factors that need to be determined before considering the use of a biomaterial are: will the material be immediately rejected or attacked by the body, will the material cause tumors to form in the surrounding tissue, and does the material cause the formation of clots [11]. Since the last factor is the main focus when finding anticoagulation coatings, the other two are the main issues that could arise.

## Anticoagulation Coatings

### Coatings that improved existing devices

Cardiovascular stents are one of the many implantable devices that improved anticoagulation coatings could greatly benefit. These devices are inserted into clogged arteries and hold them open to allow blood to flow freely. Currently, the two most commonly used types of stents are bare metal stents, which are expandable meshes made of different kinds of metals/alloys, and drug-eluting stents, which use the same mesh material, but are coated with a biodegradable polymer that contains anticoagulation and antiproliferative agents that are released into the blood over time as the polymer coating breaks down [12].

Drug-eluting stents are considered safer than bare metal stents since it has been proven that they carry a lower risk of clots forming due to the stent. However, clots do still form over time inside drug-eluting stents, because even when combined with double antiplatelet therapy, which further helps to prevent clotting, the coatings used on these stents only last a few months before they are completely dissolved [13,14]. In order to minimize the chance that thrombosis occurs, the coatings for these stents need to either break down over a much longer period of time or be able to deliver the drug without completely dissolving the polymer.

### Polymeric coatings

One of the most common methods of preventing clots over the last several decades has been the use of heparin, which is an excellent anticoagulant drug that is also good at controlling immune defense and cell growth. Effective anticoagulation coatings can be made by grafting or ionically attaching heparin to the surface of a polymeric film, and they have been shown to reduce thrombus formation by over 70% [12]. However, while heparin can make an excellent anticoagulant, it does have a few drawbacks. While it is a great anticoagulant, it also thins the blood, which can increase the risk of severe bleeding. Also, frequent use of it can cause heparin-induced thrombocytopenia (HIT), which is a prothrombotic disorder and can actually increase the risk of clots forming around the device it is coating [15].

For these reasons, heparin-mimicking and heparin-free agents have begun to be explored, especially for dialysis procedures that rely on the drug. To do this, synthetic polymers were created that contained plentiful carboxyl or sulfate groups, which are believed to be the reason for heparin’s excellent anticoagulant potential. Wang et al. synthesized one of these heparin-mimicking polymers using methylacrylic acid (MAA) and ethylene glycol diacrylate (EGDA) and used it to coat a polylactide (PLA) membrane. They found that the coated membranes had displayed decreased platelet adhesion and prolonged clotting times [15].

A group of polymers that have been widely looked at for anticoagulant coating applications are polyurethanes. Chi et al. [16] purchased a standard polyurethane film and modified it with 2-methacryloyloxyethyl phosphorylcholine (MPC). This modified film showed promising results, with increased hydrophilicity, improved platelet adhesion resistance, and they recovered almost no plasma calcium during clotting trials, even after repeated tests. However, they could not explain why this occurred. If the results of the clotting tests are accurate, then this modified polyurethane film has incredible anticoagulation properties [16].

In another trial utilizing a modified polyurethane, graphene and tricalcium phosphate (TCP) were introduced during the synthesis of a polymer consisting of polyethylene glycol (PEG), 4,4′-diisocyanate diphenylmethane (MDI), and 1,4-butanediol (BDO) as a chain extender to be used as a coating on titanium plates. While the testing for this coating was more directed at its antibacterial properties, it was shown that introducing 4 wt% of graphene into the polyurethane made it cytotoxic, making it useless for its intended purpose. Using 0.25 wt% graphene did not have this effect during the testing but may need to be explored further if this combination is to be utilized for coatings on implantable devices [17].

Another polymer-based coating option was explored by Amoako et al. [18] who attempted to “create an artificial endothelium by coupling anti-fouling zwitterionic functional groups, similar to those expressed by endothelial cells, with anti-platelet nitrous oxide, which is released by endothelial cells.” They believed that combining these methods would be equivalent to adding the effectiveness of each method alone, and the results of their testing proved their hypothesis, as the reduction of platelet adsorption was over 90% [18].

Combinations of these methods or attempts to improve them are among the various ways that improvements for these anticoagulation coatings have been explored. Several companies have looked into non-eluting heparin coatings, while others have taken the recent interest in combining graphene oxide and other anticoagulant materials as a coating for titanium, including attaching heparin to the grapheme [19,20]. However, these do not solve the problems of other similar coatings such as the possibility of heparin use leading to HIT or graphene oxide’s possible cytotoxicity if used in large quantities (Table 1).

### Non polymeric coatings

Others have looked towards different types of potential coatings not based on a polymeric film. One promising possibility is the use of metal−phenolic/catecholamine coatings. When coated onto a stainless-steel rod, the metallic coating performed well and showed good anticoagulant, antibacterial, and anti-inflammatory properties. The anticoagulant property of the coating is due to the catalytic release of nitrous oxide and while the coating was able to continuously produce nitrous oxide during testing, it was not mentioned how long this reaction may last. Because of that, this type of coating may not be as effective over time [21].

A similar coating was developed with multifunctional intent. A combination of hyaluronic acid and polydopamine, the purpose of this film was to have good characteristics in terms of anticoagulation, antihyperplasia, anti-inflammation and endothelialization, which together can be contradictory processes [22]. While the coating succeeded in showing its potential for covering all of these processes, it suffers from the same issue as the previous coating where its anticoagulant properties may rely on the release of nitrous oxide, which won’t last indefinitely.

One of the most interesting and promising non-polymeric anticoagulation coatings explored recently was the use of ultrananocrystalline diamond (UNCD). While seemingly difficult to produce, it is an extremely smooth and low-cost coating that has displayed incredible potential in terms of its anticoagulation ability. When compared to pyrolytic carbon, which is the standard material in mechanical heart valves, both displayed thrombin generation levels similar to a control, and UNCD has far better mechanical properties than pyrolytic carbon [23,24]. The only possible downfall so far is that it seems to be difficult to manufacture, despite being low cost for a diamond coating.

### Surface modifications to coatings

When considering any material for use as an anticoagulation coating on an implantable device, one of the most important aspects to consider is the surface properties of the material, since it will be making direct contact with the blood. Properties of the surface include the hydrophobicity of the material, protein adsorption, formability, and several others that can be changed in a variety of ways, including plasma treatment, chemical vapor deposition, and grafting. Regardless of the material in consideration for use as an anticoagulation coating, appropriate surface modifications need to be assessed in order to maximize the coating’s effectiveness [24–28].

## Conclusions and Future Directions

Each coating listed has its own advantages and disadvantages, and several appear to be feasible options for use as anticoagulation coatings on biomaterials. In our opinion, despite these possible few that have displayed good potential, most of these coatings are not long-term solutions. Those incorporating heparin can cause major issues over time, and while nitrous oxide can be catalyzed continuously, there’s no guarantee that the reaction won’t eventually be interrupted. Therefore, there is still a definite need to further explore anticoagulation coatings that could prevent thrombosis for long periods of time so that devices wouldn’t require repair or replacement every few years. For future direction, we propose looking towards more permanent solutions, such that implantable devices covered by one of these coatings can prolong clotting indefinitely, or at least to the point that the coated device will outlast the patient without failing due to the body’s natural reaction to foreign objects.

## Figures and Tables

**Table 1:** Summary of anticoagulation coatings.

Coating	Materials	Anticoagulantion Mechanism	Pros	Cons	[Ref]
Drug-eluting stent coating	Polymeric	Drug release	Proven effectiveness	Only lasts a few months before dissolving	[12–14]
Heparin-coated film	Polymeric	Heparin	Very effective	Increases bleeding risk, chance of developing HIT	[12,15]
Heparin-mimicking/Heparin-free coating	Polymeric	Materials with plentiful carboxyl or sulfate groups	Effective, low health risk	Not as effective as heparin due to lack of knowledge for heparin’s effectiveness	[15]
Polyurethane films	Polymeric	Surface properties	Very effective, highly modifiable	Not enough research for this type yet, room for improvements	[16]
Polyurethane/Graphene/TCP coating	Polymeric	N/A	Antibacterial properties, good mechanical properties	Becomes cytotoxic at 4 wt% graphene	[17]
Artificial endothelium	Polymeric	Zwitterionic, nitrous oxide (NO) release	Very effective	NO release may not last	[18]
Heparin/Graphene coating	Polymeric	Heparin	Effectiveness of heparin and antibacterial properties of graphene	Possibility for cytotoxicity, bleeding risk, and development of HIT	[19,20]
Metal−phenolic/ Catecholamine coating	Non-polymeric	Nitrous oxide release	Anticoagulant, antibacterial, and anti-inflammatory properties	NO release may not last	[21]
Hyaluronic acid/ Polydopamine coating	Non-polymeric	Nitrous oxide release	Anticoagulant, antihyperplasia, and anti-inflammatory properties	NO release may not last	[22]
Ultrananocrystalline diamond coating	Non-polymeric	Surface properties	Very effective, low cost, good mechanical properties	Seemingly difficult to produce	[23,24]
